# Delayed sleep timing and circadian rhythms in pregnancy and transdiagnostic symptoms associated with postpartum depression

**DOI:** 10.1038/s41398-020-0683-3

**Published:** 2020-01-21

**Authors:** Jessica L. Obeysekare, Zachary L. Cohen, Meredith E. Coles, Teri B. Pearlstein, Carmen Monzon, E. Ellen Flynn, Katherine M. Sharkey

**Affiliations:** 1 Department of Psychiatry and Human Behavior, Alpert Medical School of Brown University, 700 Butler Drive, Providence, RI 02906 USA; 2 Butler Hospital, 345 Blackstone Boulevard, Providence, RI 02906 USA; 3 Department of Psychiatry, University of North Carolina at Chapel Hill, 101 Manning Drive, Chapel Hill, NC 27514 USA; 4 Department of Psychology, Binghamton University (SUNY), Binghamton, NY 13902 USA; 5 The Women’s Medicine Collaborative, Lifespan, 146 West River Street, Providence, RI 02904 USA; 6 Department of Medicine, Alpert Medical School of Brown University, 110 Elm Street, Providence, RI 02903 USA; 7 Sleep for Science Research Laboratory, 300 Duncan Drive, Providence, RI 02906 USA

**Keywords:** Physiology, Human behaviour, Depression

## Abstract

Later sleep timing, circadian preference, and circadian rhythm timing predict worse outcomes across multiple domains, including mood disorders, substance use, impulse control, and cognitive function. Disturbed sleep is common among pregnant and postpartum women. We examined whether sleep timing during third trimester of pregnancy predicted postpartum symptoms of mania, depression, and obsessive-compulsive disorder (OCD). Fifty-one women with a previous, but not active, episode of unipolar or bipolar depression had symptoms evaluated and sleep recorded with wrist actigraphy at 33 weeks of gestation and 2, 6, and 16 weeks postpartum. Circadian phase was measured in a subset of women using salivary dim light melatonin onset (DLMO). We divided the sample into “early sleep” and “late sleep” groups using average sleep onset time at 33 weeks of gestation, defined by the median-split time of 11:27 p.m. The “late sleep” group reported significantly more manic and depressive symptoms at postpartum week 2. Longer phase angle between DLMO and sleep onset at 33 weeks was associated with more manic symptoms at postpartum week 2 and more obsessive-compulsive symptoms at week 6. Delayed sleep timing in this sample of at-risk women was associated with more symptoms of mania, depression, and OCD in the postpartum period. Sleep timing may be a modifiable risk factor for postpartum depression.

## Introduction

### Sleep disturbances in the peripartum

Women’s sleep is affected by pregnancy as early as the first trimester (for a review of sleep patterns in the peripartum, see refs. ^1,2^). As pregnancy progresses, self-reported sleep quality worsens, with decreases in reported sleep duration and sleep efficiency^3^. In addition, over 40% of pregnant women endorse insomnia in the first trimester, and this increases to over 60% by the third trimester^4^. Polysomnographic data from the third trimester has also shown increased wake after sleep onset and decreased rapid eye movement (REM) sleep compared to non-pregnant women^5^. There is acute sleep loss seen on the nights preceding and following the birth, regardless of time of day of the birth or manner of delivery^6^. Following the birth, sleep quality continues to deteriorate, with worsened sleep efficiency^7^ and further sleep loss at night, although some studies show relatively stable total sleep duration with the inclusion of scattered daytime naps^8^. Although the sleep duration may be intact, fragmentation of sleep can still cause significant daytime sleepiness in the postpartum^9^. By the end of the so-called “fourth trimester,” that is, first 3 months postpartum, the mothers’ sleep has generally begun to improve^7^, but does not necessarily return to pre-pregnancy levels^10^.

### Sleep disturbances in psychiatric disorders

The association between sleep disturbances and psychiatric illness, including depression, anxiety, mania, and psychosis, in the non-peripartum population is well established (for reviews, see refs. ^11–14^). Even in healthy individuals, acute sleep deprivation is associated with onset of mood disturbance and eventually psychotic features can emerge as sleep deprivation extends^15^. Circadian dysregulation also has been implicated in psychiatric disorders (for a review, see ref. ^16^), including associations with chronotype and later sleep timing. For instance, greater “eveningness,” that is, being a night owl, is prognostically worse from a psychiatric standpoint^17,18^. The underlying mechanisms are not fully understood, but may include alterations in cognitive^19^, psychomotor^20^, or emotional processing^21^, which in turn contribute to symptoms in a variety of psychiatric disorders.

### Peripartum psychiatric symptoms and sleep

There is overlap between the sleep alterations of pregnancy and the sleep alterations found in psychiatric disorders. Depression, mania, and obsessive-compulsive disorder (OCD) are particularly important disorders in perinatal women because they occur frequently in this population^22,23^, commonly overlap^24^, and are related to suicide in new mothers^25^. For example, sleep disturbances found both in pregnancy and depression include decreased sleep quality and sleep efficiency and increased sleep onset latency^26^. According to a 2015 Cochrane review, there is evidence for the association of self-reported poor sleep in the peripartum and postpartum depression (PPD), but the evidence for the association with peripartum anxiety and peripartum psychosis is not robust enough to be conclusive^27^. Women with bipolar disorder who reported previous manic episodes triggered by sleep loss are more than twice as likely to experience postpartum psychosis than women with bipolar disorder who never had episodes triggered by sleep loss^28^, suggesting that there may be a subgroup of women who are particularly vulnerable to perinatal sleep disturbance. The data on OCD and obsessive-compulsive (OC) symptoms and sleep disturbance in the peripartum is limited. In a non-peripartum population, sleep disturbance has been significantly associated with OCD^29,30^. Additionally, there is an increased prevalence of delayed sleep-wake phase disorder in people with severe OCD than in the general population^31^. Research on sleep in pregnant and postpartum women and its relationship to psychiatric symptoms is limited and has focused mostly on self-reported sleep data and on sleep duration rather than sleep timing. Additionally, the literature is more robust regarding depressive symptoms; less information is available for transdiagnostic manic and OC symptoms.

### Circadian rhythms and sleep as a foundation for biobehavioral regulation

In this study, we collected longitudinal, objectively measured sleep and circadian measures, including actigraphically estimated sleep onset and sleep offset times, sleep efficiency and sleep duration, circadian phase (measured with salivary dim light melatonin onset, DLMO), and relationship between sleep and circadian rhythms (defined as phase angle between salivary DLMO and sleep onset,) as well as transdiagnostic psychiatric symptoms. We conceptualize circadian rhythms and sleep as a foundation for biobehavioral regulation, with subsequent influences on many higher-order symptoms and disorders. Given the preponderance of sleep disturbance in a wide variety of psychiatric disorders^32^, it is worthwhile to study this topic in a transdiagnostic fashion. Furthermore, as the peripartum is a time of high frequency and amplitude of psychiatric symptoms^33,34^, a transdiagnostic approach to studying psychiatric symptoms in postpartum women is particularly salient. From the perspective of the Research Domain Criteria (RDoC)^35^, we are examining intersections among the following domains: Arousal and Regulatory Systems, Negative Valence Systems, and Cognitive Systems. Our goal is for this approach to begin to refine the phenotypes associated with sleep disturbance and manifestations of psychiatric illness, particularly in the peripartum. Based on the rich literature demonstrating associations between evening chronotype and poorer mental health outcomes^17,18^, we hypothesized that later sleep timing and circadian phase position during pregnancy—operationalized by sleep onset time and time of salivary melatonin onset—would be associated with greater manifestation of psychiatric and psychologic symptoms.

## Materials and methods

### Participants

The present analyses are derived from the final data set of a study with two previous, interim publications^36,37^. We analyzed data from 51 participants in whom actigraphy data were available from pregnancy. This sample size was selected based on our ability to enroll and retain participants from this difficult-to-recruit population. Post hoc power calculations showed that we maintained ~73% power to detect large effect sizes at a two-tailed *α* = 0.05. We recruited pregnant women with a past history of major depression or bipolar disorder; they underwent an informed consent process with signed consent, and received monetary compensation for participating. To be included, women had to be between the ages of 18 and 40 years and meet the Diagnostic and Statistical Manual of Mental Disorders, 4th Edition (DSM-IV) criteria for history of major depressive disorder (MDD) or bipolar disorder (I or II), diagnosed by the Structural Clinical Interview of DSM-IV Disorders^38^. We used the following exclusion criteria: current mood episode at 33 weeks of gestation (calculated by last menstrual period), diagnosis of an Axis I disorder other than an anxiety disorder (e.g., current substance dependence), a primary sleep disorder, employment as a shift worker, use of sedatives for insomnia, high-risk pregnancy, and/or plan for the infant to have a nighttime caregiver other than the participant and her partner. Antidepressant use, parity, or plan to breast or bottle feed did not exclude women from participating. The study was approved by institutional review boards at Rhode Island Hospital and Women & Infants Hospital and was carried out in accordance with the Declaration of Helsinki.

### Protocol and measurements

#### Sleep

Participants had their sleep recorded for 1 week at each of 4 time points: 33 weeks of gestation, and at 2, 6, and 16 weeks postpartum. Sleep was measured objectively using a wrist actigraph (Octagonal Basic or Micro Motionlogger Watch, activity counts measured in 1 min epochs using zero crossing mode, AMI, Ardsley, NY), worn continuously during each week. The actigraphy data were analyzed with a validated Action-W software algorithm^39^. We derived the following sleep measures from actigraphy: estimated sleep onset time, sleep offset time, and minutes of sleep. Participants recorded subjective sleep data concomitantly on a sleep diary, which was used to assist with actigraphy scoring.

#### Circadian phase

We measured DLMO phase during the last day of the 33rd week of gestation and the 6th week postpartum. Saliva was collected by participants at home, every 30 min from ~2.5 h before to ~3 h after predicted DLMO phase^40^ using Salivettes (Sarstedt, Nümbrecht, Germany). Participants wore dark glasses (Uvex, Smithfield, RI) throughout saliva collection and were contacted by a research assistant at each sample time. Samples were collected the next day, centrifuged, frozen, and stored at −20 °F, and later assayed for melatonin using radioimmunoassay (Alpco, Salem, NH at Solid Phase, Portland, ME) with a sensitivity of 0.9 pg/ml, intra-assay coefficient of variation of 7.9%, and inter-assay coefficient of variance of 11.7%. Samples were not run in duplicate and were only replicated if an error was suspected by the laboratory performing the assay. We computed DLMO phase by linear interpolation between the times of saliva samples before and after the melatonin levels reached the threshold for melatonin onset, defined as 4 pg/ml. Phase angle was defined as the difference in time (measured in hours) between actigraphically estimated sleep onset and melatonin onset.

#### Transdiagnostic features

Chronotype was measured at study entry, using the Horne–Östberg Morningness–Eveningness Questionnaire^41^ to assess evening vs. morning circadian phase preference. This is a 19-item validated questionnaire that separates participants into five chronotype categories: definite morning type, moderate morning type, definite evening type, moderate evening type, or neither type. In our sample, the Morningness-Eveningness Questionnaire had a Cronbach’s *α* of 0.71.

We evaluated psychiatric symptoms at the end of each study week during a visit to the participant’s home. Participants completed the Highs scale for mania^42^, which asks patients to identify if they have experienced manic symptoms in the past 3 days via a 7-item questionnaire. The Obsessive-Compulsive Inventory (OCI^43^), which assesses for OCD symptomatology using 42 questions rated on a Likert scale, and includes 7 subscales (e.g., washing, checking), was administered at 33 weeks of pregnancy and 6 weeks postpartum. This scale had a Cronbach’s *α* of 0.95 at 33 weeks of gestation and 0.96 at 6 weeks postpartum. The 17-item Hamilton Rating Scale for Depression (HAM-D17^44^) was completed by a trained clinician at the end of each study week; Cronbach’s *α* for this scale was 0.64 at 33 weeks, 0.74 at 2 weeks postpartum, 0.69 at 6 weeks, and 0.84 at 16 weeks.

#### Statistical analyses

We used SPSS Version 19 (IBM, Chicago, IL). For these analyses, we divided the participants into an “early sleep” group (*n* = 25) and a “late sleep” group (*n* = 26), based on median split of sleep onset time during the third trimester. Data are described using mean and standard deviation. We used two-tailed tests throughout, including repeated-measures analysis of variance (ANOVA) to test for differences in sleep measures between groups and across the four study time points and *t* tests to make post hoc comparisons. We did not adjust for multiple comparisons.

## Results

### Participants

Fifty-one women were included in these analyses with a mean age of 28.2 ± 5.2 years (range: 18–38 years). Forty-four had a past history of MDD (86.3%) and seven had a past diagnosis of bipolar disorder. Eighteen (35.3%) were first-time mothers and the median number of children among experienced mothers was 1 with a range of 1–4. Fifteen women reported taking at least one medication with the potential to affect sleep or mood during the third trimester, including antidepressants (*n* = 6), benzodiazepines (*n* = 1), levothyroxine (*n* = 1), pain relievers, specifically acetaminophen (*n* = 5), β-blockers (1), inhaled β-agonists (1), anti-histamines, specifically ranitidine and loratadine (*n* = 4), and/or anti-emetics (*n* = 1). The sample was ethnically, racially, and socioeconomically diverse. The majority of women (70.6%) were “neither” types on Horne–Östberg Morningness–Eveningness Questionnaire. Six (11.8%) were “moderate morning” types and nine (17.9%) were “moderate evening” types. See Table 1 for additional participant demographics.

**Table 1:** Demographics: collected by self-report at the time of study enrollment, *n* = 51.

	Total *n* = 51 *n* (%)
Self-reported race/ethnicity	
White/Caucasian	32 (62.7%)
Hispanic/Latina	7 (13.8%)
Multiracial	5 (9.8%)
Black/African-American	3 (5.9%)
Other	3 (5.9%)
Asian	1 (2%)
Reported involvement with partner/husband/father of baby	
Yes	42 (82.4%)
No	9 (17.6%)
Reported working outside the home or attending school at study enrollment (third trimester)	
Yes	28 (54.9%)
No	23 (45.1%)
History of trauma exposure (reported by patients on the PTSD section of the SCID)	
Yes	36 (70.6%)
No	15 (29.4%)
Horne–Östberg Morningness–Eveningness Questionnaire category	
Definite morning	0
Moderate morning	6 (11.8%)
Neither	36 (70.6%)
Moderate evening	9 (17.9%)
Definite evening	0

### Sleep measures

Average actigraphically estimated sleep onset time during the third trimester (pregnancy week 33) ranged from 9:01 p.m. to 1:58 a.m., with a median of 11:27 p.m. To test our hypotheses about associations between delayed sleep timing and transdiagnostic psychiatric symptoms, we used the median sleep onset time of 11:27 p.m. during the third trimester of pregnancy to split the sample into two groups: “early sleep” and “late sleep.” Repeated-measures ANOVA revealed that, as expected, there was a main effect of group in sleep onset time (*F* = 43.214, *p* < 0.001). Sleep offset time also differed significantly between groups (*F* = 17.163, *p* < 0.001), with group differences averaging more than 1 h at all four study weeks. Although the average Horne–Östberg Morningness–Eveningness scores were in the “neither” range for both groups, they differed significantly between groups (53.9 vs. 44.9, *t* = 5.4, *p* < 0.001), with the late group showing more evening preference. Furthermore, all six moderate morning types were in the “early sleep” group and all nine moderate evening types were in the “late sleep” group. Most women fell into the same group at all three postpartum time points (week 2: 68.6%; week 6: 76.4%; week 16: 72.9%). Antidepressant use was divided evenly between groups; three women in the “early sleep” group reported taking sertraline. In the “late sleep” group, one woman took sertraline, one took nortriptyline, and one took bupropion and clonazepam.

Maternal sleep measures for “early sleep” and “late sleep” groups at 33 weeks of gestation, and 2, 6, and 16 weeks postpartum are shown in Table 2. As would be expected across the perinatal period, a main effect of study week was observed for sleep offset time (*F* = 9.784, *p* < 0.001), time in bed (*F* = 6.007, *p* = 0.002), sleep duration (*F* = 7.727, *p* < 0.001), and sleep efficiency (*F* = 28.605, *p* < 0.001). Within-subject contrasts revealed significant differences between sleep duration and sleep efficiency at postpartum weeks 2 and 6 compared with pregnancy, and significant differences in sleep offset time and time in bed between postpartum weeks 2 and 16 compared with pregnancy. Finally, we observed significant week by group interactions for sleep onset time (*F* = 4.316, *p* = 0.009), time in bed (*F* = 3.451, *p* = 0.024) and sleep duration (*F* = 3.34, *p* = 0.028). Planned post hoc *t* tests showed that sleep onset differed significantly between the “early sleep” and “late sleep” groups at all four study weeks, whereas the interaction effects for time in bed and sleep duration between groups were driven by significant differences only during week 33 of pregnancy. During pregnancy, the “late sleep” group spent less time in bed (8.0 vs. 8.7 h, *t* = 2.920, *p* = 0.005) and had shorter estimated sleep durations (6.4 vs. 7.3 h, *t* = 2.693, *p* = 0.01) than the “early sleep” group.

**Table 2:** Sleep, circadian measures, and transdiagnostic symptoms from 33 weeks of gestation to 16 weeks postpartum.

Measure	33 Weeks		2 Weeks		6 Weeks		16 Weeks	
	Early	Late	Early	Late	Early	Late	Early	Late
Sleep and circadian measures								
Sleep onset (clock time ± minutes)	10:38 p.m. ± 35	12:33 a.m. ± 46*	11:07 p.m. ± 56	12:10 a.m. ± 65*	11:11 p.m. ± 62	12:17 p.m. ± 62*	10:58 p.m. ± 53	12:13 a.m. ± 69*
Sleep offset (clock time ± min)	7:31 a.m. ± 64	8:29 a.m. ± 78*	7:43 a.m. ± 59	9:03 a.m. ± 82*	7:49 a.m. ± 64	8:52 a.m. ± 73*	7:04 a.m. ± 55	8:00 a.m. ± 79*
Time in bed (min)	524 ± 41	477 ± 68*	517 ± 54	534 ± 65	507 ± 74	516 ± 56	488 ± 56	468 ± 75
Estimated sleep duration (min)	439 ± 73	385 ± 71*	369 ± 62	372 ± 52	384 ± 57	375 ± 57	406 ± 53	379 ± 66
Sleep efficiency (%)	84.5 ± 11.0%	82.0 ± 7.6%	73.0 ± 7.1%	71.3 ± 7.1%	77.6 ± 6.9%	74.1 ± 7.5%	84.4 ± 6.3%	81.9 ± 7.6%
DLMO (clock time ± min)	9:05 p.m. ± 74	9:46 p.m. ± 88			9:31 p.m. ± 80	10:05 p.m. ± 93		
Phase angle (h)	1.5 ± 1.0	2.7 ± 1.2*			1.6 ± 1.2	2.0 ± 1.1		
Transdiagnostic symptoms								
Highs	1.40 ± 1.63	2.19 ± 2.0	0.92 ± 1.32	2.31 ± 2.09*	1.16 ± 1.70	1.88 ± 2.29	1.33 ± 1.95	2.00 ± 1.84
OCI	14.28 ± 19.75	18.37 ± 21.01			14.00 ± 21.98	18.04 ± 23.45		
HAM-D17	5.88 ± 3.11	6.69 ± 3.58	6.08 ± 3.00	9.35 ± 5.61*	6.96 ± 3.98	9.04 ± 5.15	6.79 ± 4.18	9.21 ± 8.26

Sleep measures were collected by actigraphy during each study week, and circadian measures were determined using salivary dim light melatonin onset measured at week 33 of pregnancy and week 6 postpartum. Hypomanic/manic symptoms were measured with the Highs scale. Obsessive-compulsive symptoms were measured with the Obsessive-Compulsive Inventory (OCI). Depressive symptoms were measured with the 17-item Hamilton Rating Scale for Depression (HAM-D17)

*p < 0.05, showing when the “late sleep” group was significantly different compared with the “early sleep” group

### Dim light melatonin onset

Melatonin onset data were available for 45 participants at 33 weeks of pregnancy and 37 participants at 6 weeks postpartum. Clock time of DLMO did not differ significantly between “early sleep” and “late sleep” groups (see Table 2), but pregnant women in the “late sleep” group had significantly longer phase angles between DLMO and sleep onset time during pregnancy than the early group (2.7 ± 1.2 vs. 1.5 ± 1 h, *t* = −3.487, *p* = 0.001), indicating that they were going to sleep later with respect to their internal circadian clock. Although phase angle did not differ between groups at 6 weeks postpartum, women in the “late sleep” group had a significant shortening of their phase angle from the third trimester to 6 weeks postpartum (*t* = 2.371, *p* = 0.024), whereas phase angle did not change in the “early sleep” group. On average, “late sleep” mothers’ sleep onset time occurred 55 min earlier with respect to circadian phase at 6 weeks postpartum than during pregnancy.

### Transdiagnostic psychiatric symptoms: associations with sleep timing and melatonin onset

The results of the transdiagnostic psychiatric symptoms are shown by study week in Table 2.

### Hypomanic/manic symptoms (Highs scale)

**Fig. 1:**
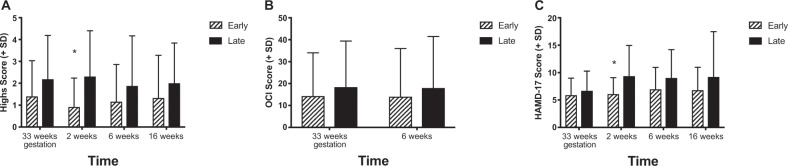
Transdiagnostic psychiatric symptoms. This figure shows a comparison of transdiagnostic symptoms scores between “early sleep” and “late sleep” groups. **a** The results for the Highs scale, which measured hypomanic/manic symptoms at each study week and was significantly different at week 2. **b** The Obsessive-Compulsive Inventory (OCI), which was collected at week 33 of gestation and 6 weeks postpartum. **c** The Hamilton Rating Scale for Depression (HAM-D17), which showed that women in the “late sleep” group had more depressive symptoms at 2 weeks. *p < 0.05.

**Fig. 2:**
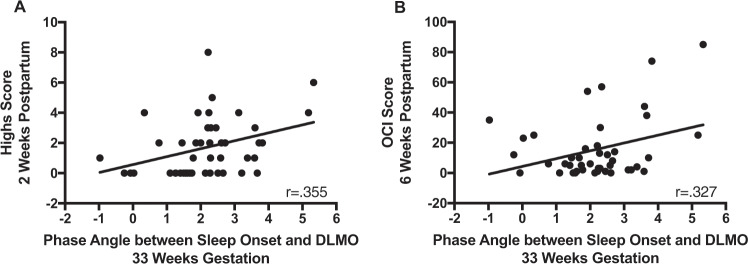
Phase angle and psychiatric symptoms. The phase angle is calculated as the difference between the time of sleep onset (measured by actigraphy) and the time of dim light melatonin onset (DLMO, measured with salivary melatonin), with a wider phase angle indicating a greater lag between melatonin onset and the participant’s sleep onset time. **a** A significant positive correlation (*p* = 0.018) between third trimester phase angle and manic/hypomanic symptomatology measured at 2 weeks postpartum. **b** A significant positive correlation (*p* = 0.032) between third trimester phase angle and obsessive-compulsive symptoms at 6 weeks postpartum. This indicates that a later bedtime (relative to internal circadian clock) during the third trimester of pregnancy is associated with increased hypomanic/manic and obsessive-compulsive symptoms in the early postpartum.

There was an overall effect of group (*F* = 5.494, *p* = 0.024), but no significant effect of time and no time × group interaction on the Highs scale. Although Highs scores were higher in the “late sleep” group at all weeks (Fig. 1a), this difference was statistically significant only at postpartum week 2 (*t* = −2.786, *p* = 0.008), with the “late sleep” group showing significantly more manic symptoms. Highs score at 2 weeks also was correlated significantly with longer phase angle at 33 weeks (*r* = 0.355, *p* = 0.018; Fig. 2a), consistent with the notion that delayed sleep timing with respect to circadian phase in pregnancy is associated with symptoms such as racing thoughts, difficulty concentrating, and feeling more active than usual in the early postpartum period.

### Obsessive thoughts/compulsions (OCI)

OCI score did not differ significantly between the early and late sleep groups at 33 weeks of pregnancy or 6 weeks postpartum (Fig. 1b), however, OCI score at 6 weeks postpartum was correlated with longer phase angle at 33 weeks of pregnancy (*r* = 0.327, *p* = 0.032, Fig. 2b), indicating that women who went to sleep later with respect to circadian phase during third trimester showed more OC symptoms at 6 weeks postpartum.

### Depressive symptoms (HAM-D17)

ANOVA showed no effect of time and no group × time interaction on depressive symptoms measured by the HAM-D17, but the main effect of group approached significance (*F* = 4.033, *p* = 0.051). Post hoc *t* tests showed that compared with the “early sleep” group, the “late sleep” group had significantly higher HAM-D17 scores at postpartum week 2 (*t* = −2.576, *p* = 0.013, Fig. 1c). Longer phase angle at 33 weeks did not correlate significantly with HAM-D17 scores.

## Discussion

This study showed that among a population that is notorious for poor sleep—pregnant women—certain aspects of sleep confer an increased risk of postpartum psychiatric symptoms in women at risk for PPD. Later sleep in pregnancy impacted domains that are relevant across diagnoses, indicating the foundational influence of sleep behavior across RDoC constructs. Specifically, later sleep timing in third trimester was associated with shorter sleep duration during pregnancy and predicted greater manic and depressive symptoms in the early postpartum weeks. This study split women with a past history of depression or bipolar disorder into an “early sleep” and “late sleep” group based on sleep timing during pregnancy. Notably, there were no differences in psychiatric symptoms during pregnancy when the differences in sleep timing and duration were observed. Rather, more psychiatric symptoms were observed postpartum in the “late sleep” group. Sleep onset and offset times estimated with wrist actigraphy continued to be significantly delayed in the “late sleep” group during all three postpartum time points, but the groups’ sleep duration and sleep efficiency did not differ postpartum as both groups experienced shortened and disrupted sleep as has been well described among postpartum women due to infant care^2^.

Our measure of circadian phase, clock time of DLMO, did not vary significantly between groups, but the “late sleep” group had significantly longer phase angles between sleep onset and DLMO during pregnancy, implying that the women in the “late sleep” group were staying up later with respect to their internal circadian timing. Interestingly, the phase angles of women in the “late sleep” group shortened significantly from pregnancy to the postpartum period, whereas phase angle remained stable (and shorter) in the “early sleep” women. One explanation for this observation is that increased homeostatic drive for sleep in the postpartum period allowed the “late sleep” women to fall asleep earlier with respect to their internal clock. Women with longer phase angle in pregnancy were significantly more likely to have increased manic symptoms at 2 weeks postpartum and OC symptoms at 6 weeks postpartum.

Later sleep timing during pregnancy was associated with more manic symptoms at 2 weeks postpartum as measured on the Highs scale. Although these symptoms are sometimes described as elation and may overlap with the normal/expected happiness associated with the arrival of a new baby, in our sample, the most commonly reported symptoms were suggestive of subtle cognitive impairment, namely racing thoughts and difficulty concentrating. As manic symptoms in the very early postpartum days have been associated with later onset of PPD^45^, one interpretation is that the observed elevation in the Highs scale is an early sign of cognitive disruption resulting from sleep disruption.

Our study adds to the evidence that sleep changes in pregnancy are associated with depressive symptoms in the postpartum^46,47^. Although none of the women in this at-risk sample were in a mood episode at third trimester, women with sleep onset later than 11:27 p.m. during pregnancy were rated clinically by a psychiatrist as significantly more depressed at 2 weeks postpartum. Prior research has shown that pharmacological treatment of insomnia during pregnancy resulted in reduced postpartum depressive symptoms^48^. The current study raises the question of whether prescribing earlier sleep timing during pregnancy could also prevent PPD.

Sleep timing in pregnancy was not related to postpartum OC symptoms in this sample, as has been shown in patients with OCD^31^. On the other hand, longer phase angle at third trimester predicted more OC symptoms at 6 weeks postpartum. None of the women in this sample met the criteria for OCD at study entry, but OC symptoms are transdiagnostic, particularly in perinatal women, where they can overlap with anxiety about the wellbeing of the infant^49^. Our observed association between OC symptoms and later sleep onset with respect to circadian timing is consistent with the notion that phenomena such as repetitive/intrusive thoughts and impairment in response-inhibition may make it more difficult for women to fall asleep, highlighting the bidirectionality of delayed sleep timing and psychiatric symptoms. We speculate that increased OC symptoms may be particularly troublesome at night when the increasing homeostatic drive for sleep may lead to disinhibition that allows for manifestation of OC behaviors and thoughts.

Limitations of our study include our small, heterogeneous sample and the use of multiple measures and statistical comparisons. We have published other papers from sub-samples of this data set^36,37^ and acknowledge that interim analyses can introduce bias, although the focus of those earlier publications differed substantially from the present report, which is the first paper that includes the whole sample. We did not control for medication use in our analyses, but note that antidepressant use was divided evenly across the two groups at 33 weeks. None of the women in this high-risk sample met the criteria for a depressive episode at the 33-week assessment, but it is possible that some participants may have been at even higher risk due to medication discontinuation that we did not measure. Interestingly, both women taking medications currently considered less conventional in pregnancy (i.e., nortriptyline, bupropion/clonazepam) were both in the “late sleep” group, but they would not be expected to delay sleep onset time as prescribed.

In addition, we recognize the potential limitations of categorizing our participants into “early sleep” and “late sleep” groups rather than using sleep onset time as a continuous variable, including loss of power to detect small and/or non-linear associations between delayed sleep timing and postpartum psychiatric and psychologic symptoms. Nevertheless, we chose this strategy intentionally because of the clinical salience of identifying a recommended pregnancy bedtime cutoff, after which, there could be an increased risk of postpartum symptoms. While we acknowledge that an individual woman with a sleep onset of 11:25 p.m. likely does not differ meaningfully from a woman with a sleep onset of 11:30 p.m., tying the concept of how later sleep timing may translate to increased psychiatric risk to actual clock times may serve to translate our findings into clinical practice.

This study also adds to the growing evidence that later chronotype is associated with transdiagnostic psychiatric symptoms in other populations. For example, a recent prospective study of middle-aged women showed a significant trend of later chronotype with development of depressive symptoms^50^, and a study of young adults including patients receiving treatment for unipolar depression and controls found associations between circadian timing and depression severity, as well as the presence of hypomanic symptoms^51^.

In conclusion, our analyses indicate that late sleep timing in pregnancy—as measured by clock time and with respect to women’s internal circadian rhythms—confers a risk of increased psychiatric symptoms across several cognitive, behavioral, and regulatory domains. Sleep habits in late pregnancy represent a potential target for future treatment studies, for a goal of reduction in transdiagnostic psychiatric symptoms in the postpartum period.
